# Inhibitory, Anticancer Efficacy and Structural Insights of a Novel Ethyl 1‐Hydroxy‐3‐Oxo‐1‐Phenylisoindoline‐2‐Sulfonate Compound Selectively Targeting Human CA IX and CA XII Carbonic Anhydrase, Ferroptosis‐Mediated Cell Death

**DOI:** 10.1002/cbf.70336

**Published:** 2026-11-02

**Authors:** Funda Keteci, Songul Bayrak, Ufuk Atmaca, Mustafa Özkaraca, Azada Aliyeva, Buket Bakan

**Affiliations:** ^1^ Department of Molecular Biology and Genetics Atatürk University Erzurum Turkey; ^2^ Department of Chemistry, Faculty of Sciences Atatürk University Erzurum Turkey; ^3^ Department of Pathology, Faculty of Veterinary Medicine Cumhuriyet University Sivas Turkey

**Keywords:** biochemistry, carbonic anhydrase, cell death, enzyme inhibition, isoindolinone

## Abstract

Carbonic anhydrase (CA) isoforms IX and XII are critical in anticancer drug development due to their essential roles in regulating pH and hypoxic conditions. In this study, the inhibitory potential of ethyl‐1‐hydroxy‐3‐oxo‐1‐phenylisoindoline‐2‐sulfonate (1), synthesized via click chemistry, was evaluated against hCA IX and hCA XII. The cellular effects of compound on human breast cancer (MCF‐7 cells) were investigated using a combination of viability, oxidative stress, and molecular level. Compound exhibited stronger inhibitory activity than the acetazolamide (AAZ), with *K*_i_ values of 11.48 ± 4.18 nM for hCA IX and 9.32 ± 2.35 nM for hCA XII. The compound exhibited a higher binding affinity to hCA IX with −8.8 kcal/mol docking score. While no cytotoxic effect was observed on MCF‐10A cells, a significant dose‐dependent reduction was observed in cell viability, confirmed loss of membrane integrity in cancer cells. ROS levels were decreased at higher concentrations, indicating that the toxicity was independent of ROS production. *Caspase‐3* and *LC3B* expression showed strong positivity at moderate concentrations, suggesting activation of apoptotic and autophagic pathways. *GPX4* and *TFRC* fluorescence intensities were markedly decreased that disruption of redox homeostasis and iron metabolism. These findings indicate that compound is a promising lead molecule for the development of next‐generation anticancer agents targeting tumor‐associated CA isoforms.

## Significance Statement

Ethyl 1‐hydroxy‐3‐oxo‐1‐phenylisoindoline‐2‐sulfonate strongly inhibited the tumor‐associated carbonic anhydrases hCA IX and hCA XII at nanomolar concentrations and exhibited greater inhibitory potency than acetazolamide under the applied cell‐free assay conditions. In human breast cancer cells (MCF‐7), the compound produced concentration‐dependent anticancer activity and modulated markers associated with apoptotic, autophagic, and ferroptosis‐related pathways. Comparative evaluation in nontumorigenic mammary epithelial cells (MCF‐10A) were contributed to assessing its preliminary safety profile. Together, the potent inhibition of tumor‐associated CA isoforms and the observed cellular responses support the compound as a promising scaffold for further anticancer drug‐development studies. Considering the hypoxia‐dependent activity of CA IX in aggressive breast cancer, future evaluation in models under hypoxic conditions would broaden the translational relevance of the present findings.

## 1 Introduction

Heterocyclic compounds, particularly indoles, constitute a fundamental class of scaffolds in medicinal chemistry owing to their natural occurrence in biomolecules such as tryptophan and serotonin, as well as in numerous plant alkaloids [1]. Structural isomers of the indole scaffold, namely isoindolinone derivatives, have gained substantial research interest in recent years due to their structural diversity, suitability for diverse functionalization, and broad spectrum of pharmacological activities. The aromatic isoindolinone core represents a key structural motif in various biologically active molecules, including alkaloids, phthalocyanines, fused heterocycles, isoindoline‐based pigments, and isoindole–BODIPY dyes, all of which are associated with a wide range of biological effects [2, 3, 4].

Carbonic anhydrase enzymes (CAs, EC 4.2.1.1) catalyze the reversible hydration of CO_2_ to bicarbonate and protons, thereby playing essential roles in various physiological processes, including pH regulation, electrolyte balance, respiration, modulation of metabolic pathways, bone resorption, and tumor progression [5, 6]. The heterogeneous distribution of human CA isoforms has highlighted the importance of developing isoform‐specific inhibitors, which represent a strategic objective spanning drug discovery to clinical applications. In particular, the membrane‐bound isoenzymes CA IX and CA XII, which are strongly associated with tumor hypoxia, have emerged as desirable therapeutic targets due to their minimal expression in normal tissues and their marked overexpression in hypoxic solid tumors [7, 8]. Inhibition of CA IX/XII has been shown to suppress tumor microenvironment acidification, reduce tumor cell proliferation, and enhance the sensitivity of cancer cells to existing chemotherapeutic agents, underscoring the value of these isoforms as a promising avenue in the design of anticancer drugs [9]. Breast cancer is a significant global health challenge, requiring the development of novel treatment strategies with clearly defined mechanisms of action [10]. Overcoming therapeutic limitations in breast cancer remains a major difficulty, highlighting the need to explore novel molecular targets.

In this context, isoindolinone derivatives, owing to their structural flexibility and favorable target‐interaction profiles, represent promising molecular scaffolds for developing inhibitors targeting tumor‐associated CA isoforms. In the present study, the inhibitory activity of ethyl 1‐hydroxy‐3‐oxo‐1‐phenylisoindoline‐2‐sulfonate against isolated hCA IX and hCA XII was evaluated together with its molecular docking profile. Its cellular effects were examined in MCF‐7 estrogen receptor‐positive luminal breast cancer cells by assessing viability, membrane integrity, oxidative stress, and cell‐death‐associated markers. In addition, a comparative evaluation in a nontumorigenic mammary epithelial cell line was performed to assess the compound's preliminary safety profile.

## 2 Materials and Methods

### 2.1 Materials

WST‐1 reagent was obtained from Takara Bio Inc. (Shiga, Japan). CA IX/XII isoenzymes and all other chemicals were purchased from Sigma‐Aldrich. All solvents, 2‐benzoylcarboxylic acid, and alcohols used in this study were commercially available and utilized without further purification. IR spectra were recorded using a Perkin‐Elmer spectrophotometer with 0.1 mm cells in CHCl3.

### 2.2 Chemistry

#### 2.2.1 General Procedure Synthesis of Ethyl‐1‐Hydroxy‐3‐Oxo‐1‐Phenylisoindoline‐2‐Sulfonate

Chlorosulfonyl isocyanate (1.1 eq) was introduced to a solution of 2‐benzoylbenzoic acid (1.0 eq) and a catalytic quantity of trifluoroacetic acid in 10 mL of dichloromethane, and the mixture was agitated at ambient temperature for 2 h. Then, 1 mL of the corresponding alcohol (ROH) was added and stirring was maintained at room temperature for 1 h. Subsequent to the reaction, volatiles were extracted under reduced pressure. The resulting residue was purified via thin‐layer chromatography using a mixture of ethyl acetate and *n*‐hexane (1:4) as the eluent, yielding the pure product [11].

### 2.3 Carbonic Anhydrase Inhibitory Effect Study

To determine the inhibitory effects of the compound ethyl 1‐hydroxy‐3‐oxo‐1‐phenylisoindoline‐2‐sulfonate (1), the esterase activities of the hCA IX and hCA XII isoforms were analyzed with the Verpoorte method [12], which measures absorbance changes at 348 nm [13, 14]. The compound Isoindolinone (1) and the reference inhibitor AAZ were dissolved in DMSO at an initial concentration of 1 mg/mL. At the end of the reaction mixture, the DMSO concentration was approximately 1%. As in previous studies, the activities of the hCA isoforms were evaluated using 4‐nitrophenyl acetate (PubChem CID: 13243) as the substrate. The quantity of enzyme needed to catalyze the conversion of 1 μmol of substrate per minute at 25°C was established as one enzyme unit. Every sample was examined in triplicate.

To investigate the in vitro mechanisms of inhibition of Isoindolinone (1), kinetic experiments were performed with different substrate and inhibitor concentrations. The resulting data were used to construct Michaelis–Menten curves and Lineweaver–Burk plots, as well as to calculate *K*_i_ values and determine the type of inhibition [15].

### 2.4 Cell Culture and Anticancer Activity Assay

MCF‐10A (human nontumorigenic epithelial cell line, ATCC‐CRL‐10317) and MCF‐7 (human breast cancer cell line, ATCC HTB‐22) were cultured in DMEM/high glucose medium with containing of 10% fetal bovine serum, 100 U/mL penicillin/streptomycin, at 37°C, 5% CO_2_. All cellular treatments were conducted under standard normoxic culture conditions; no chemical or chamber‐based hypoxia induction was applied. The cells were free of mycoplasma contamination for the described experiments and actively proliferating in the logarithmic phase in earlier passage number were used in the experiments. The assessment of cell viability was conducted using in vitro water‐soluble tetrazolium salt (WST‐1) and the lactate dehydrogenase (LDH) assay [16]. The amount of 3 × 10^4^ cells/mL were cultured in 96‐well plates and after 24 h, the cultured cells were exposed with varying concentrations of compound (6.25–500 μg/mL) for 24 h. Cell morphologies were observed under inverted microscopy and optical density was recorded at 450 nm in a Thermo microplate reader.

In WST‐1 assay, the cell viability was calculated as formulated below:

(%) Viable cells = ([(the absorbance of the treated cells) − (the absorbance of the blank)])/([(the absorbance of the control) − (the absorbance of the blank)) × 100.

In the LDH assay, the absorbance variation was measured at 340 nm at intervals of 15 s over a duration of 3 min. Cell viability was calculated according to the following formula:

Slope of lysate/(slope of lysate + slope of supernatant) × 100 and relative viability as (sample viability/untreated control viability) × 100.

### 2.5 Measurement of Oxidative Stress (ROS) Level

The oxidative stress levels were determined by using fluorescent molecular probes, which based on the oxidation of the dye 2′,7‐dichlorofluorescein (DCFH‐DA) and dihydroethidium (DHE) by different reactive oxygen species (ROS) [17]. DCFH‐DA easily penetrates cell membranes and is hydrolyzed by cellular esterase to yield DCFH, which is then oxidized to generate the fluorescent molecule 2′,7‐dichlorofluorescein (DCF) upon exposure to hydroxyl radicals. DHE oxidizes to produce bright 2‐hydroethidium (EOH) upon exposure to a superoxide radical, facilitating precise monitoring of DHE fluorescence without the risk of interconversion fluctuation [18]. ROS formation was conducted on MCF‐7 cells treated with test sample at 6.25–500 μg/mL concentrations, followed by incubation at 37°C for 4 h. While untreated cells were used as the negative control, hydrogen peroxide (100 μM H_2_O_2_) served as the positive control. After treatment, the cells were washed three times with phosphate buffer saline (PBS) to eliminate interference of dye, and thereafter stained with 20 μM DCFH‐DA and 20 μM DHE for 30 min at 37°C. Data were shown as percentage fluorescence relative to the corresponding negative controls.

### 2.6 Immunofluorescence Staining

To determine the cell death mechanism, immunohistochemistry study was performed on breast cells [16]. Cells were fixed with methanol for 5 min at −20° C and washed with PBS. Then, the cells were incubated with PBS including 0.1% Triton X‐100 for 15 min at room temperature. After the washing step, the cells were incubated with PBS containing 2% BSA for 60 min at room temperature. Subsequent to re‐washing, the cells were treated with polyclonal anti‐Caspase‐3 (Thermofisher, Catalog No. PA5‐114687), monoclonal anti‐LC3B (Santa Cruz, Catalog No. sc‐271625), polyclonal anti‐GPX4 (BT Lab, Catalog No. BT‐AP02883), and monoclonal anti‐TFRC (BT Lab, Catalog No. BT‐MCA2826) primary antibodies at a 1/200 dilution at +4°C overnight. Cells were washed with PBS again and incubated with goat anti‐mouse FITC (Jackson ImmunoResearch, Catalog No. 115‐095‐003) and goat anti‐rabbit FITC (Jackson ImmunoResearch, Catalog No. 111‐095‐003) at a 1/50 dilution for 1 h at room temperature in the dark. Finally, 4′,6‐diamidino‐2‐phenylindole (DAPI) was dropped onto the cells, and the cells were examined under a fluorescence microscope (Zeiss Axio). The intensity of fluorescence positivity in the cells was evaluated using the Fiji ImageJ program.

### 2.7 Molecular Docking Study

The structure of the compound was drawn using ChemDraw 20.0 and saved in SDF format (https://www.perkinelmer.com). The 3D molecular structures of the Human Carbonic Anhydrase IX (PDB ID: 6FE2) and Human Carbonic Anhydrase XII (PDB ID: 8CO3) were retrieved from the Protein Data Bank (https://www.rcsb.org) [19]. Protein structure preparation was performed using UCSF Chimera 1.14. For both hCA IX (PDB ID: 6FE2) and hCA XII (PDB ID: 8CO3), all crystallographic water molecules, co‐crystallized solvent molecules, and noncatalytic heteroatoms were systematically removed prior to docking simulations. Importantly, the catalytic Zn^+2^ ion located within the active site pocket was strictly retained to maintain the essential coordination architecture of the enzymes. Standard polar hydrogens were added, and appropriate protonation states were assigned to ensure structural integrity. Ligand preparation was carried out using UCSF Chimera 1.14 following initial 2D‐to‐3D structure conversion from ChemDraw 20.0. Standard hydrogen atoms were added to assign appropriate protonation states corresponding to physiological pH (7.4). Partial atomic charges were assigned using the Gasteiger charge model. To obtain a stable, low‐energy 3D conformation free of steric clashes, the ligand structure underwent energy minimization using standard force field parameters prior to docking simulations. For comparative evaluation, the standard reference carbonic anhydrase inhibitor, acetazolamide (AAZ; PubChem CID: 1986), was retrieved from the PubChem database and prepared using identical protonation and energy minimization parameters. Then, the molecular docking analyses of Human Carbonic Anhydrase IX and Human Carbonic Anhydrase XII with compound ligand and AZZ were performed by CB‐DOCK server (http://clab.labshare.cn/cb-dock/php/blinddock.php) [20], which combines curvature‐based cavity detection with the AutoDock Vina docking algorithm. To ensure full reproducibility, the search space and grid parameters were defined based on the primary catalytic cavities. For hCA IX (PDB ID: 6FE2), the grid box was centered at coordinates *X* = −35, *Y* = −20, *Z* = −5 with box dimensions of 20 × 20 × 27 Å^3^. For hCA XII (PDB ID: 8CO3), the grid box center was established at coordinates *X* = −12, *Y* = −18, *Z* = 22 with dimensions of 20 × 20 × 20 Å^3^. The analysis of the docking results was carried out using Discovery Studio Visualizer [21]. Finally, the protein‐ligand docked structure was shown and colored by the Chimera 1.14 visualization tool [22].

### 2.8 Statistical Analysis

To analyze the differences between the groups, one‐way ANOVA and Duncan multiple comparison test were performed with *p* values indicated by *p ≤ 0.05, **p ≤ 0.01, and ***p ≤ 0.001 by using GraphPad Prism software, and the results were expressed as the mean ± standard deviation (SD). Immunofluorescence staining data were analyzed using SPSS 20.0 program using one‐way‐ANOVA and post hoc Tukey test. The significance level was accepted as *p* < 0.001.

## 3 Results and Discussion

### 3.1 Carbonic Anhydrase Inhibition Results

Due to the considerable similarity among the active regions of different hCA isoforms, the tailing technique has become a highly promising approach for the design and synthesis of selective hCA inhibitors [23, 24]. This approach involves incorporating aryl or heterocyclic moieties into the aromatic sulfonamide ring to selectively interact with hydrophobic or hydrophilic residues located in the outer region of the isoform‐specific active site [25].

Carbonic anhydrase isoforms IX and XII have emerged as priority therapeutic targets in the development of next‐generation anticancer agents, as they are markedly overexpressed in the hypoxic tumor microenvironment, play a central role in regulating pH homeostasis in tumor cells, and sustain an acidic extracellular environment that promotes metastatic potential [26, 27].

In this study, the inhibitory activity of ethyl 1‐hydroxy‐3‐oxo‐1‐phenylisoindoline‐2‐sulfonate (1) was evaluated against the tumor‐associated hCA IX and hCA XII isoforms. Compound **1** exhibited IC_50_ values of 12.15 nM for hCA IX and 13.07 nM for hCA XII, with corresponding *K*_i_ values of 11.48 ± 4.18 and 9.32 ± 2.35 nM, respectively (Table 1 and Figure 1). The same chemical compound, designated as compound **2a** in our previous study, exhibited IC_50_ values of 24.29 and 28.84 nM and *K*_i_ values of 22.03 ± 9.21 and 21.69 ± 10.56 nM against hCA I and hCA II, respectively [11]. Comparison of these values indicates an approximately 1.9–2.4‐fold preference for hCA IX and hCA XII over the cytosolic hCA I and hCA II isoforms (Table 1). Therefore, compound **1** demonstrates modest preferential inhibition of the tumor‐associated isoforms rather than strict isoform selectivity.

**Table 1:** Comparative IC_50_ and *K*_i_ values of compound against hCA I, hCA II, hCA IX, and hCA XII isoforms.

Compound	CA isoform	IC_50_ (nM)	*R* ^2^	*K*_i_ (nM)	Data source
Compound^a^	hCA I	24.29	0.9861	22.03 ± 9.21	Previous study [11]
Compound^a^	hCA II	28.84	0.9906	21.69 ± 10.56	Previous study [11]
Compound^b^	hCA IX	12.15	0.9898	11.48 ± 4.18	Present study
Compound^b^	hCA XII	13.07	0.9964	9.32 ± 2.35	Present study
AAZ^b^	hCA IX	13.96	0.9828	20.89 ± 1.72	Present study
AAZ^b^	hCA XII	15.98	0.9878	18.16 ± 0.88	Present study

Abbreviation: AAZ, acetazolamide.

^a^ The hCA I and hCA II inhibition data for the same chemical compound, designated as compound **2a** in the previous publication, were reported in Yazıcıoğlu et al. [11].

^b^ The hCA IX and hCA XII inhibition data were obtained in the present study.

**Figure 1:**
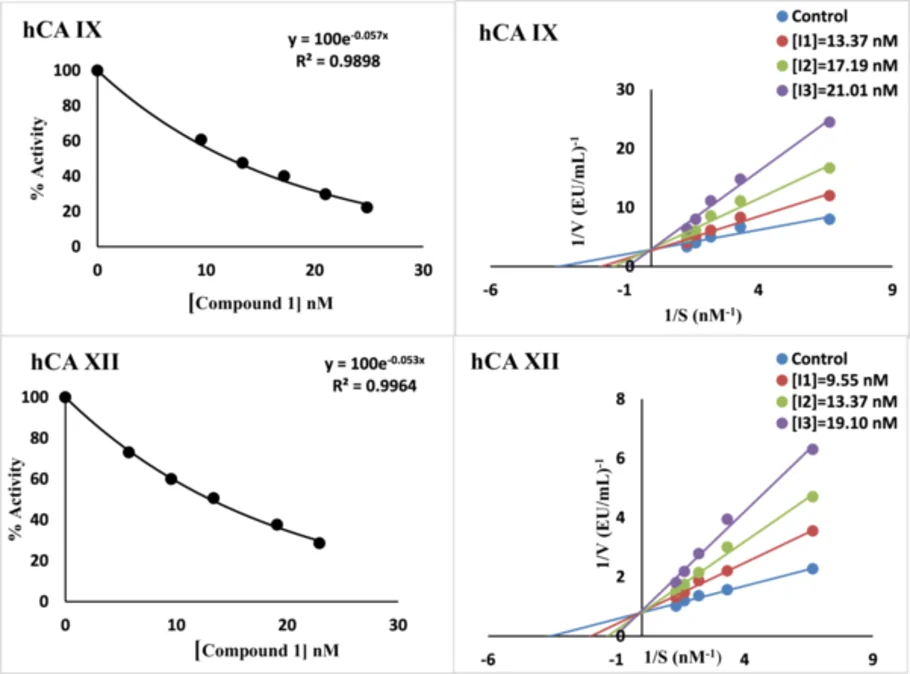
IC_50_ and *K*_ᵢ_ plots of hCA IX and hCA XII isoenzymes.

The majority of studies on indole and isoindolinone derivatives in the literature have focused on the hCA I and hCA II isoenzymes. Our research group has previously investigated the inhibitory effects of isoindolinone compounds on these isoforms [11]. Similarly, Nadaroğlu and colleagues reported the inhibitory activities of a series of isoindoline‐1,3‐dione derivatives against the hydratase functions of hCA I and hCA II [28]. Awadallah and colleagues evaluated the inhibitory potential of compounds bearing thiazolinone or imidazolone indole tails on hCA I, II, IV, and IX isoenzymes [29]. In addition, Gündoğdu and colleagues examined the effects of various substituted isoindole‐1,3‐dione sulfonamides on hCA I and hCA II [30]. Although the existing literature primarily concentrates on hCA I and hCA II, the present study offers a distinctive contribution by investigating the inhibitory profiles of the clinically relevant tumor‐associated isoenzymes hCA IX and hCA XII.

The hCA IX and hCA XII inhibition assays in the present study were performed using isolated enzymes and were therefore independent of the cellular expression levels of these isoforms. The nanomolar inhibition constants consequently demonstrate direct enzyme‐inhibitory potency but do not, by themselves, establish cellular target engagement. Nevertheless, the expression patterns of hCA IX and hCA XII provide a biological rationale for evaluating these isoforms as therapeutic targets. In particular, hCA IX exhibits restricted expression in most normal tissues but is strongly induced in hypoxic tumors, potentially providing a target‐based therapeutic advantage [31]. In contrast, hCA XII is also expressed in several normal epithelial tissues and exhibits subtype‐dependent expression in breast cancer; thus, enzyme inhibition data should be interpreted together with cellular selectivity and safety findings [32]. The hypoxia‐dependent expression and activity of hCA IX in MDA‐MB‐231 cells [33], together with its reported contribution to breast cancer stem‐cell maintenance within hypoxic niches [34], further support the potential relevance of hCA IX inhibition in aggressive and triple‐negative breast cancer. Future studies using aggressive breast cancer models under normoxic and hypoxic conditions, combined with direct measurements of cellular hCA IX/hCA XII expression and activity, will help clarify the contribution of these isoforms to the biological effects of compound **1**.

### 3.2 Cytotoxicity and Anticancer Activity

The cytotoxic effects of the compound were evaluated in nontumorigenic epithelial cells (MCF‐10A) using WST‐1 (Figure 2a) and LDH (Figure 2b) assays at concentrations ranging from 6.25 to 500 µg/mL. According to the results, cell viability was remained at low and intermediate concentrations, remaining close to the control level, whereas a slightly decrease was detected at highest concentration, reaching approximately 80% viability in both WST‐1 and LDH assays. The compound did not cause a substantial decrease in cell viability to reach the 50% inhibitory concentration (IC_50_) within the applied concentration range.

**Figure 2:**
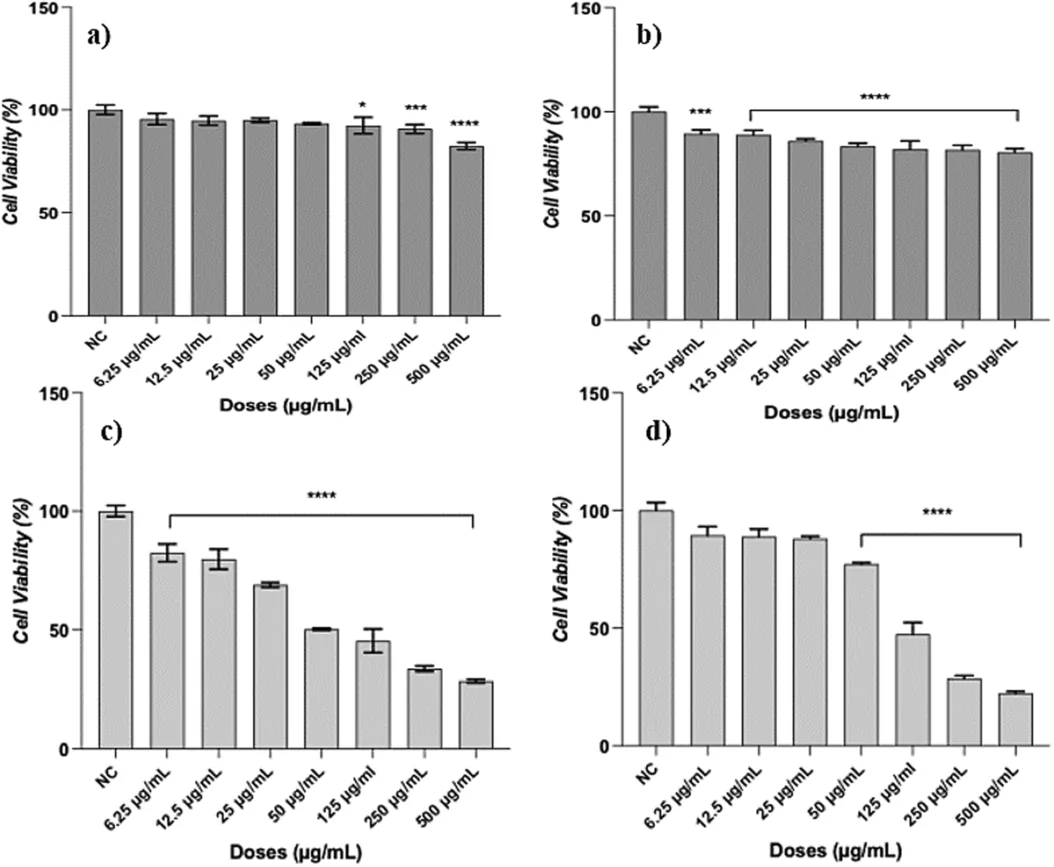
Cytotoxicity and anticancer activity results of compound in MCF‐10A (a and b) and MCF‐7 (c and d) cells after 24 h exposure by WST‐1 assay (a and c) and LDH assay (b and d). Data are presented as mean ± SD, *^,^***^,^**** indicates statistically significant difference compared the control group (NC).

The anticancer efficacy of the tested compound on MCF‐7 cells was evaluated in a concentration‐dependent manner for 24 h. As shown in Figure 2c,d, cell viability gradually decreased with increasing concentrations of the compound by WST‐1 and LDH assay, which reflect cellular metabolic activity and membrane integrity, respectively. The reduction in cell viability at higher concentrations was highly significant compared to the negative control (*p* < 0.0001), which indicated anticancer effects of compound. IC_50_ value was calculated as 86.553 µg/mL. The increase in extracellular LDH suggests a loss of plasma membrane integrity, confirming the reduction in cell viability. It may represent a potential candidate for further mechanistic and therapeutic investigations. Certain structural features and reactivity parameters can influence the biological response of substances. In the study of Mesci et al. [35], they investigated isoindole‐derived heterocycle compounds against MCF‐7 and MCF‐12A cells and they concluded that the compounds have significant antioxidant activities, and were cytotoxic in MCF‐7 cells. Tan and colleagues mentioned that isoindole derivatives exert beneficial effects on inflammatory diseases, including cancers and the influence of substituents is crucial in assessing the anticancer activity of compounds [36, 37]. The cellular experiments in the present study were conducted under normoxic conditions without hypoxia induction. The HIF‐1α pathway is primarily responsible for regulating CA IX, which is highly induced in hypoxic environments, whereas less expressed in normoxia [38]. This distinction is important because CA IX expression is generally low under normoxia and strongly induced following hypoxic exposure, whereas MCF‐7 cells may exhibit constitutive CA XII expression under normoxic conditions [39]. Therefore, although compound directly inhibited isolated hCA IX and hCA XII, the cytotoxic response observed in normoxic MCF‐7 cells cannot be attributed specifically to cellular CA IX inhibition. The cellular effects may involve CA XII inhibition, CA‐independent mechanisms, or interactions with additional molecular targets. The accompanying changes in cell‐death‐associated markers demonstrate biological activity but do not provide direct evidence of intracellular CA IX target engagement. The observed cytotoxicity may possibly be contributed to by CA IX‐independent pathways, even though our results were consistent with a possible role of CA IX inhibition. Future studies should evaluate CA IX and CA XII expression and activity under normoxic and hypoxic conditions and employ target‐validation approaches, such as genetic knockdown or pharmacological rescue, to determine the contribution of each isoform to the cellular response [33, 39, 40].

### 3.3 ROS Formation

The most extensively researched ROS in cancer cytotoxicity include hydrogen peroxide, hydroxyl radicals, and superoxide [41]. DCFDA and DHE fluorescent probes were used to assess intracellular ROS generation, which mainly identifies general oxidative stress and superoxide anion levels [16]. Low concentrations of compound did not significantly change ROS levels when compared to the negative control (NC), as shown in Figure 3a,b, suggesting that these doses did not cause oxidative stress. However, at higher concentrations (125–500 µg/mL), intracellular ROS levels were significantly reduced, as shown by reduced DCFDA and DHE fluorescent signals (*p* < 0.0001). This decrease of ROS level points to either a possible antioxidant activity of the compound at high concentrations. These results imply that ROS overproduction is not the mechanism behind the cytotoxic effects seen at high doses. Alternatively, ROS‐independent processes such as direct membrane damage and mitochondrial malfunction without ROS leakage can cause cell death [42]. Severe cytotoxicity and membrane damage may compromise cellular integrity and intracellular retention of the fluorescent probes, thereby resulting in an apparently lower fluorescence signal. In this context, the marked LDH release observed at the same concentrations supports the possibility that membrane damage and loss of cellular integrity contributed to the reduced ROS signal. Furthermore, reduced metabolic activity and a lower number of viable cells at these concentrations may also contribute to the apparent decrease in ROS‐related fluorescence.

**Figure 3:**
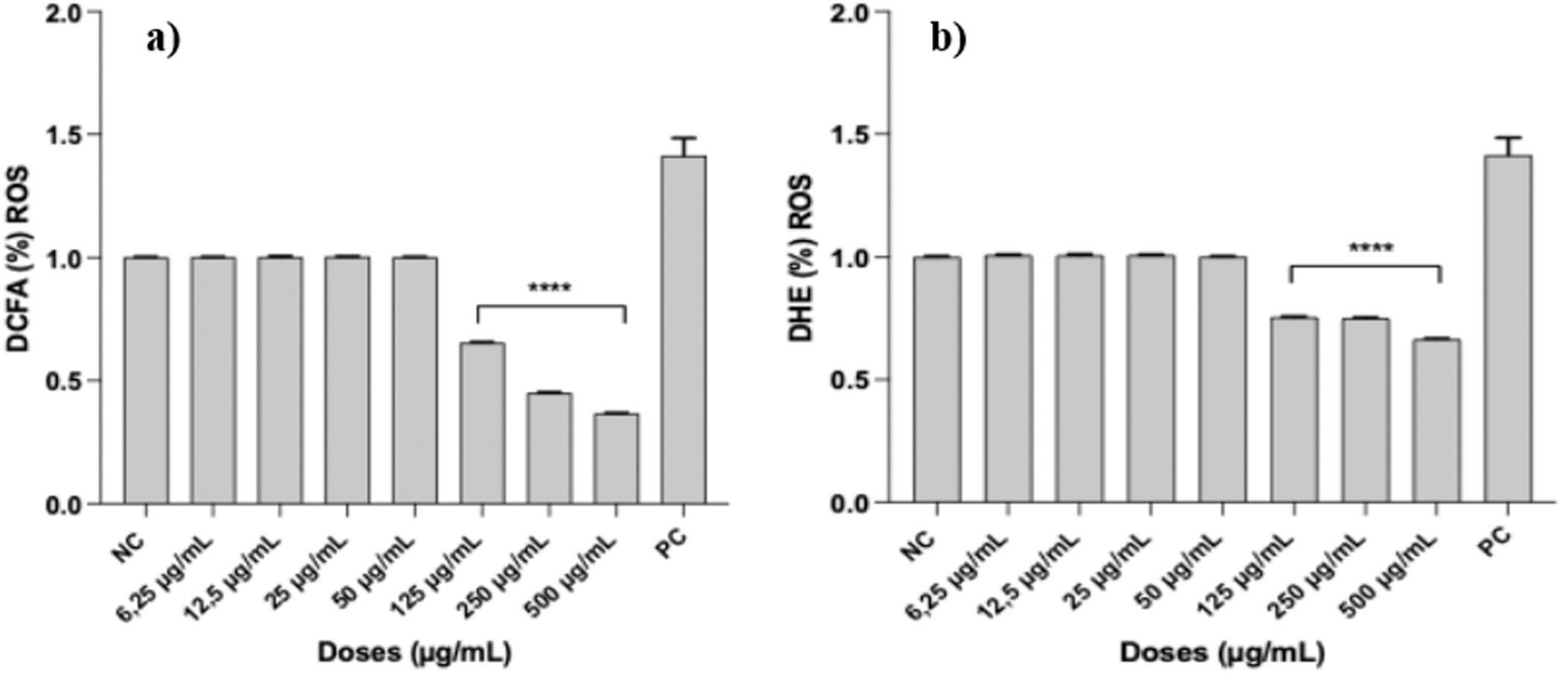
Effects of compound on intracellular ROS level in MCF‐7 cells after 4 h incubation. Cells were stained with 20 μM DCFH‐ DA and 20 μM DHE dye. NC, negative control (untreated cells), H_2_O_2_ treated groups served as positive control. Data represent the mean fluorescence signal with ±SD with calculation against the negative controls and at least 10,000 events were collected per sample (the **** indicates the difference compared with the negative control group).

### 3.4 Immunofluorescence Staining

In order to investigate cell death pathways signaling at the mechanistic level were performed on MCF‐7 cells in selected concentrations that obtained from cytotoxicity data. Statistically significant differences were found between application groups in immunofluorescence staining in terms of *Caspase‐3, LC3B, GPX4*, and *TFRC* markers (Table 2). According to the results, *Caspase‐3* and *LC3B* showed similar responses as positive effects were severe in the negative control (NC) group, 125 μg/mL, and 250 μg/mL dose groups, while mild positivity was detected in the 500 μg/mL dose group. This pattern indicated that the compound primarily activated controlled cell death processes at moderate concentrations (125 and 250 µg/mL), namely autophagy and apoptosis. The reduced *Caspase‐3* and *LC3B* positivity at the highest concentration (500 µg/mL) indicated that apoptotic and autophagic signaling pathways could be suppressed and the cells could be undergone other death pathways (Figures 4 and 5).

**Table 2:** Immunofluorescence positivity of Caspase‐3, LC3B, GPX4, and TFRC positively between treatment groups in MCF‐7 cells.

Groups	Caspase‐3	LC3B	GPX4	TFRC
NC	26.17 ± 9.28^a^	23.30 ± 7.04^a^	14.61 ± 4.28^a^	12.42 ± 3.10^a^
125 μg/mL	24.09 ± 7.71^a^	25.83 ± 6.59^a^	16.48 ± 3.09^a^	11.63 ± 4.82^a^
250 μg/mL	27.21 ± 10.08^a^	23.46 ± 8.54^a^	7.18 ± 2.54^b^	6.78 ± 3.16^b^
500 μg/mL	9.28 ± 3.51^b^	8.02 ± 4.81^b^	6.97 ± 3.30^b^	5.63 ± 2.82^b^

^a,b^ Differences between groups, *p* < 0.001.

**Figure 4:**
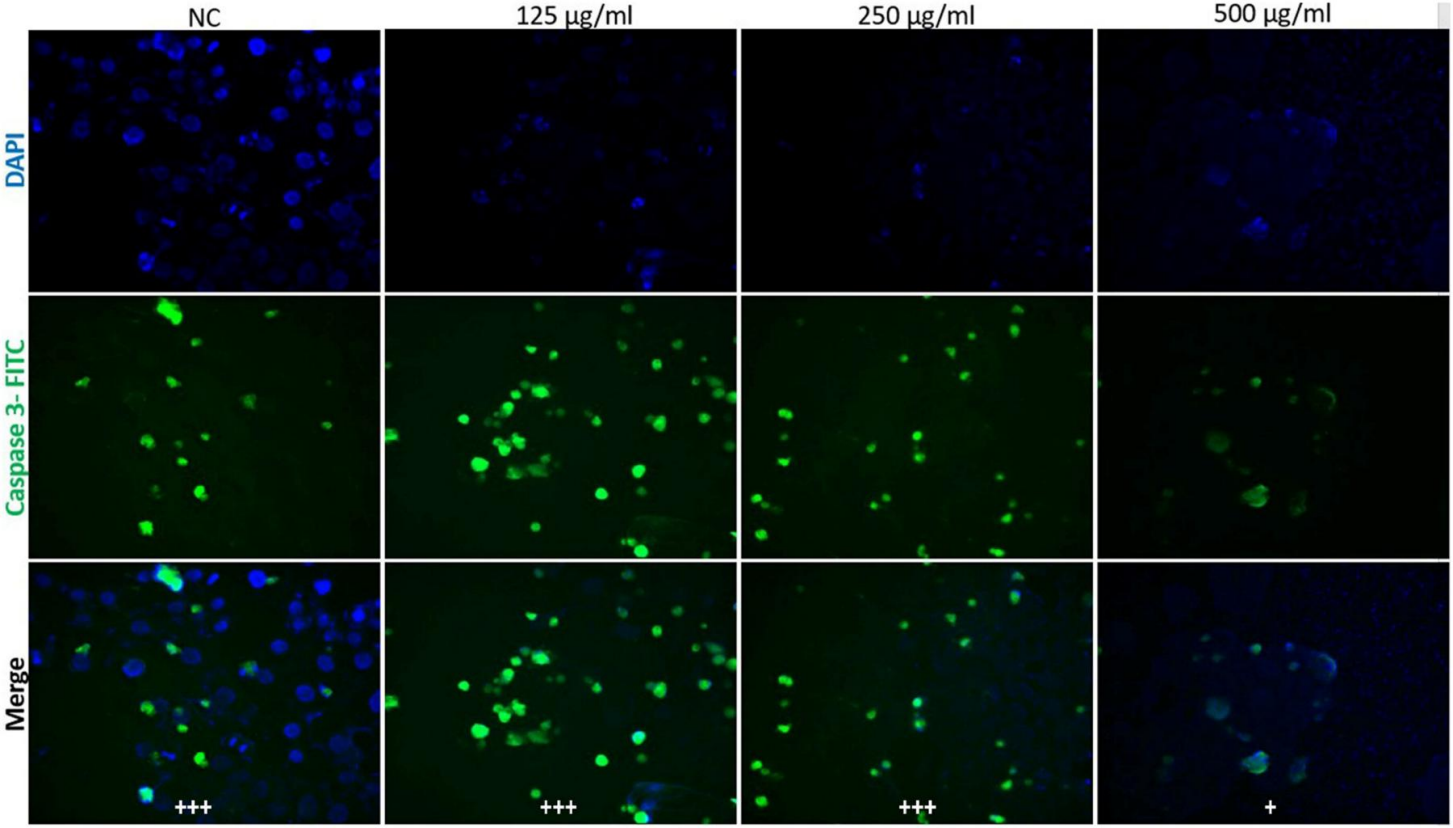
Positivity on MCF‐7 cells by immunofluorescence staining. *Caspase 3* fluorescence positivity in the *NC group* severe level, *125 μg/mL group* severe level, *250 μg/mL group* severe level, *500 μg/mL group* mild level (+ mild), (+++ severe). NC, negative control.

**Figure 5:**
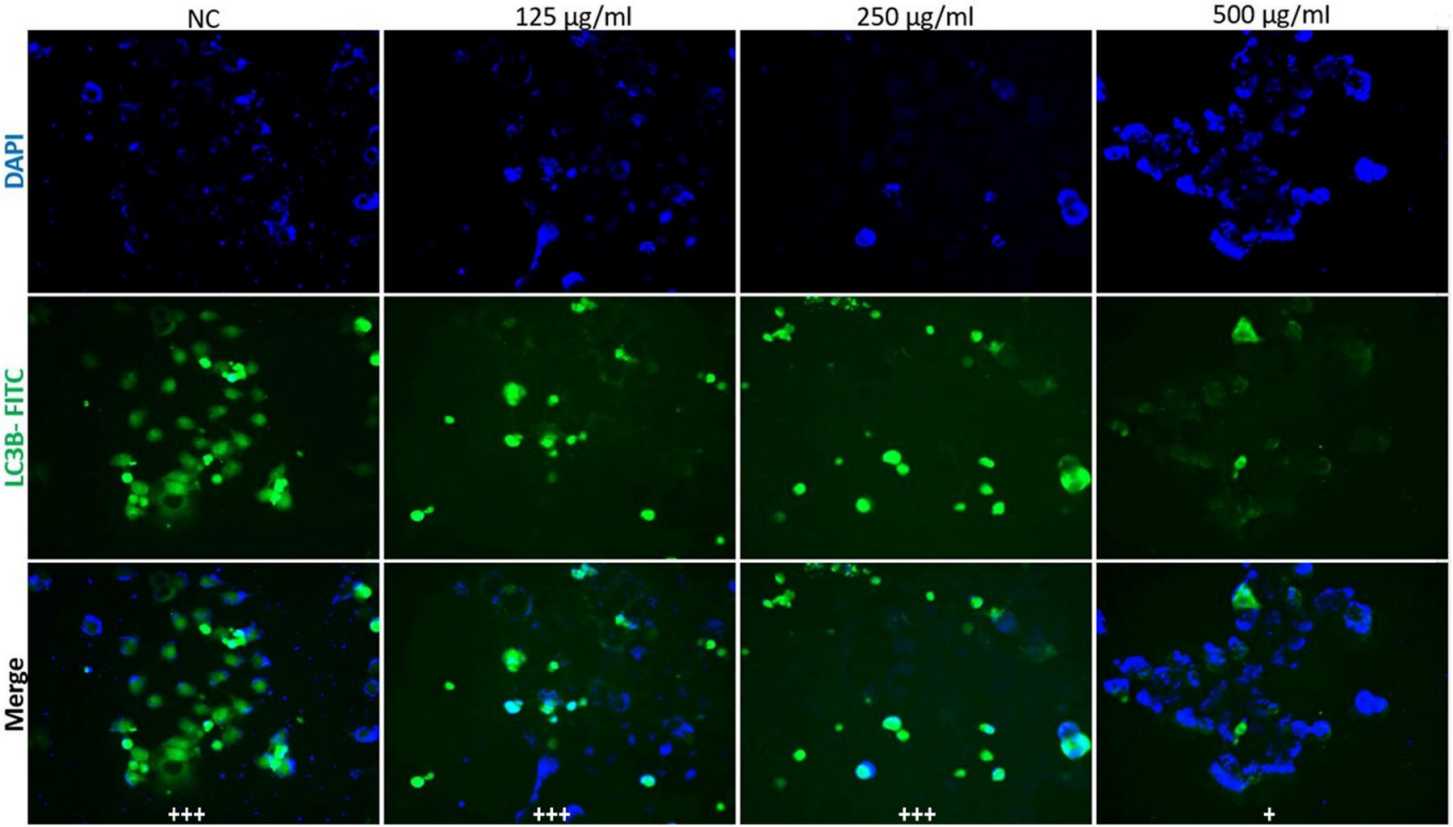
Positivity on MCF‐7 cells by immunofluorescence staining. *LC3B* fluorescence positivity in the *NC group* severe level, *125 μg/mL group* severe level, *250 μg/mL group* severe level, *500 μg/mL group* mild level (+ mild), (+++ severe). NC, negative control.

*GPX4* and *TFRC* fluorescence positivity were moderate in the NC group and 125 μg/mL dose groups, and it was a mild level in the 250 μg/mL and 500 μg/mL dose groups (Figures 6 and 7). *GPX4* is a key antioxidant enzyme that protects cells from lipid peroxidation, whereas *TFRC* is involved in iron uptake and is closely associated with ferroptosis regulation [43]. Dxion and colleagues coined the term ferroptosis in 2012 [44], which is a controlled, iron‐dependent type of cell death caused by lipid peroxidation that is very different from other types of cell death. Ferroptosis has a critical role in the development and treatment of tumors, suggesting that ferroptosis could be a therapeutic approach that successfully induces cancer cell death as a substitute for conventional therapy. Overall, these results suggested that lower to moderate concentrations (125–250 µg/mL) predominantly elicited apoptosis and autophagy, whereas higher concentrations (500 µg/mL) transition the mechanism of cell death to a more severe and less regulated process, potentially implicating ferroptosis‐related pathways. The concurrent decrease in *GPX4* and *TFRC* expression at elevated doses substantiates the concept that oxidative balance and iron regulation are disrupted, leading to irreversible cellular damage instead of regulated apoptotic signaling. Our data indicated that, for the first time, treatment with the test compound results in characteristic aspects of ferroptosis.

**Figure 6:**
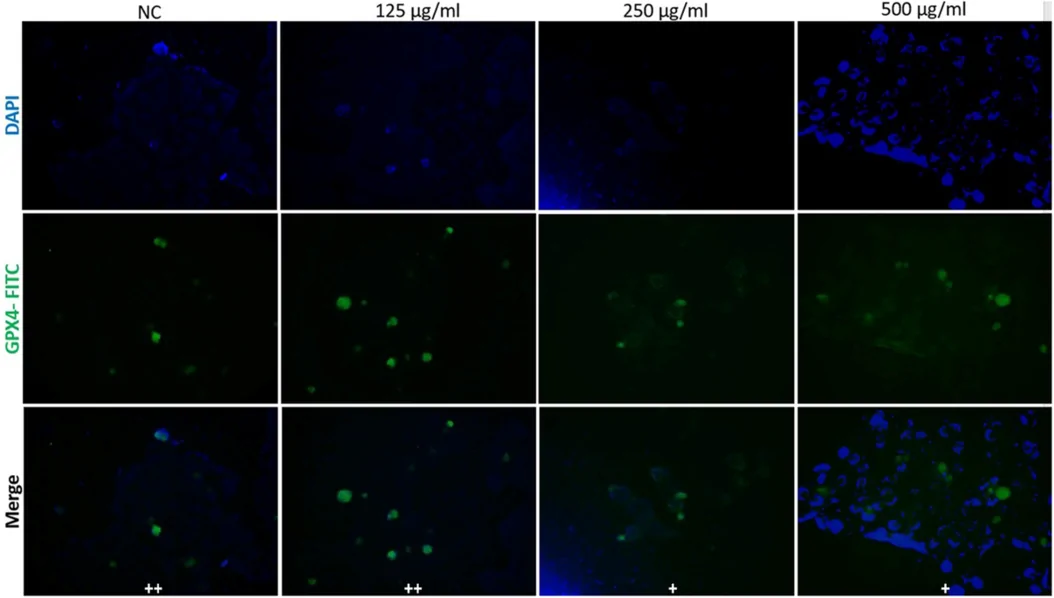
Positivity on MCF‐7 cells by immunofluorescence staining. *GPX4* fluorescence positivity in the *NC group* moderate level, *125 μg/mL group* moderate level, *250 μg/mL group* mild level, *500 μg/mL group* mild level (+ mild), (++ moderate). NC, negative control.

**Figure 7:**
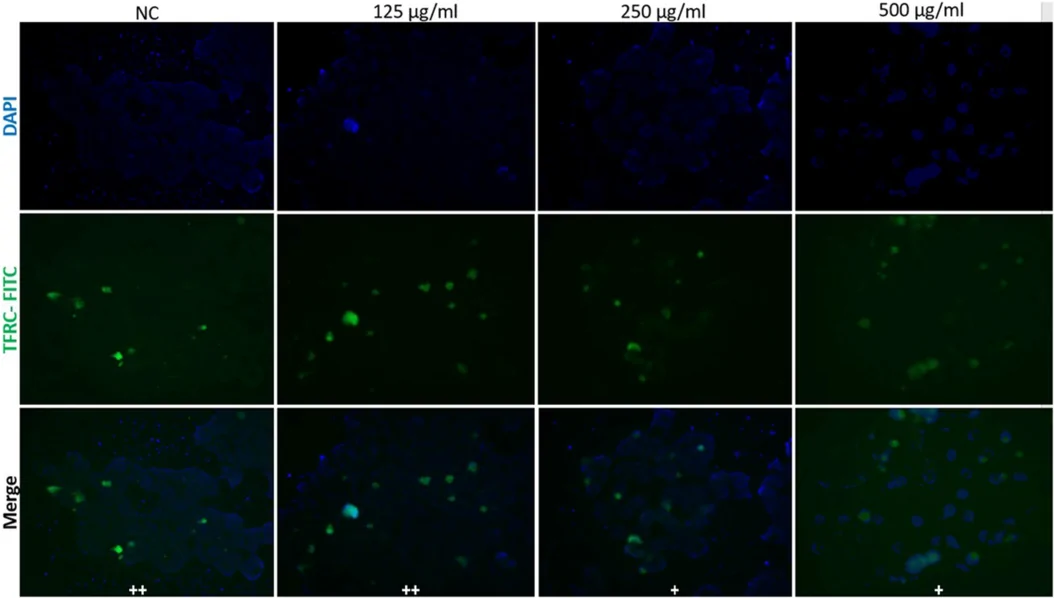
Positivity on MCF‐7 cells by immunofluorescence staining. *TFRC* fluorescence positivity in the *NC group* moderate level, *125 μg/mL group* moderate level, *250 μg/mL group* mild level, *500 μg/mL group* mild level (+ mild), (++ moderate). NC, negative control.

### 3.5 Molecular Docking

Molecular docking is widely used as an initial in silico screening approach to predict ligand–target interactions prior to experimental validation. By providing insights into binding orientation, interacting residues, and relative binding affinity, docking studies assist in rational compound selection and significantly reduce the time and cost associated with early‐stage wet‐lab experiments [45, 46]. In this context, molecular docking is considered a complementary computational tool that guides subsequent in vitro and in vivo investigations rather than replacing experimental validation [47, 48]. Isoindolinone‐ and phenyl‐substituted heterocyclic scaffolds have been frequently investigated in docking‐based studies due to their structural rigidity and ability to form stable hydrophobic and aromatic interactions within enzyme binding pockets. Previous reports have shown that phenyl‐containing isoindoline derivatives can effectively engage key residues of enzymatic targets, supporting their suitability for structure‐based drug design [49].

In the current study, Human Carbonic Anhydrase IX (hCA IX) and Human Carbonic Anhydrase XII (hCA XII) were docked with the ligand compound, standard reference inhibitor acetazolamide (AAZ), and their binding energies were calculated as presented in Table 3. Using Discovery Studio Visualizer, comprehensive 2D interaction diagrams were created to show comprehend the binding mechanism (Figure 8). A strong hydrogen‐bonding network and advantageous π‐mediated contacts were found in the compound's noncovalent interaction study within the hCA IX binding cavity (Figure 8A). In particular, the sulfonyl oxygen atoms and residues GLN249 and ARG246, as well as the ester oxygen and SER155, established typical hydrogen bonds. The complex was further stabilized by the scaffold's aromatic phenyl ring through a π–π T‐shaped contact with TRP16 and a π‐alkyl interaction with ARG246. A π‐donor hydrogen bond was also established with ALA247. The preferential binding affinity for hCA IX is explained by these cooperative noncovalent interactions. Molecular docking studies of the hCA XII revealed a very advantageous binding orientation in the active site cavity (Figure 8B). The core scaffold establishes a strong conventional hydrogen bond between the ester oxygen and GLU247, accompanied by a supportive carbon‐hydrogen bond with the same residue. Also, π‐mediated contacts, such as π–π stacking and π–π T‐shaped interactions with PHE8, significantly stabilize the aromatic framework. Furthermore, ARG246 and ILE249 generate hydrophobic π‐alkyl interactions and through a vast web of van der Waals interactions, surrounding active site residues such as PRO245, CYS237, GLU260, ASN250, and ASP259 further anchor the ligand. Docking analysis against hCA IX for the reference inhibitor acetazolamide (AAZ) showed a unique interaction profile inside the catalytic pocket (Figure 8C). ARG26 and the acetamide moiety's carbonyl oxygen create a traditional hydrogen bond, in addition to hydrophobic π‐alkyl interactions with LEU54B and ALA22, a π‐donor hydrogen bond with GLN205 stabilizes the core thiadiazole ring. Additionally, the sulfonamide group's oxygen atoms form helpful carbon–hydrogen bonds with GLY136 and ALA204. Through van der Waals interactions, surrounding residues such as PHE27, GLY25, PRO55, CYS203, GLU56, and GLN175 further stabilize the complex. Molecular docking for the reference inhibitor acetazolamide (AAZ) against hCA XII showed a large hydrogen‐bonding network maintaining the ligand inside the active pocket (Figure 8D). The acetamide group and residues GLN28 and LEU27, as well as the sulfonamide moiety and residues ASN14, GLU13, GLY9, and LYS257, form conventional hydrogen bonds. A carbon–hydrogen bond with PHE8 and a π‐sigma contact between the thiadiazole ring and LYS257 further stabilize the complex. Via van der Waals interactions, surrounding active site residues such as TYR7, GLY12, GLY25, LEU26, GLN256, PHE258, ASP259, and ARG261 offer extra structural anchoring. The results indicated that Carbonic Anhydrase IX exhibited a higher binding affinity for the compound compared to Carbonic Anhydrase XII, as supported by the interacting residues shown in Figure 9. In the present study, the compound displayed favorable docking profiles against both CA IX and CA XII, with Vina scores of −8.8 and −7.8 kcal/mol, respectively, and predicted binding within sizeable cavities (803 Å^3^ for CA IX; 636 Å^3^ for CA XII). The interaction patterns involved aromatic/polar residues (e.g., TYR7 and ARG246‐region contacts), which is consistent with the residue‐level engagement commonly discussed in carbonic anhydrase docking studies. In particular, TYR7 is located in the hydrophobic entrance to the catalytic pocket, where it stabilizes aromatic ring systems close to the active site rim and promotes lipophilic interactions. On the other hand, ARG246 is located in the hydrophilic area of the active site wall and serves as an electrostatic anchor and essential hydrogen‐bond donor for polar functional groups. Our docking models showed that TYR7 and ARG246 engage noncovalently, which is consistent with known binding modes for isoform‐selective carbonic anhydrase inhibitors and isoindolinone‐based derivatives [50, 51, 52]. While compared to the clinical reference inhibitor acetazolamide (AAZ; −6.7 kcal/mol for hCA IX and −7.6 kcal/mol for hCA XII; Table 3), the compound showed improved binding affinities for both human carbonic anhydrase isoforms, hCA IX (−8.8 kcal/mol; cavity volume: 803 Å^3^) and hCA XII (−7.8 kcal/mol; cavity volume: 636 Å^3^). The aromatic isoindolinone framework, which successfully occupies the large lipophilic active site cleft and engages in an extensive noncovalent network—including conventional hydrogen bonds with GLN249, ARG246, and SER155, as well as π–π T‐shaped stacking with TRP16, which is responsible for the target compound's strong binding performance, especially against hCA IX. In contrast, AAZ is restricted to a smaller cavity envelope (333 Å^3^) and mainly depends on localized polar contacts. Importantly, the magnitude of the predicted affinities is in line with previously reported docking ranges for CA isoforms; for instance, Haider and colleagues reported Vina‐scale binding energies around −9.8 kcal/mol for CA IX and −7.9 kcal/mol for CA XII for their evaluated derivatives [53]. Overall, these results provide a mechanistic rationale to proceed with experimental confirmation, such as CA IX/CA XII inhibition assays and isoform selectivity profiling, in accordance with standard structure‐based screening workflows [54].

**Table 3:** Binding energies (docking scores) and contact residues of compound with human Carbonic Anhydrase IX and XII.

Receptors	Ligand	Vina docking score (kcal/mol)	Cavity volume (Å3)	Contact residues
Carbonic Anhydrase IX	Compound	−8.8	803	ARG6 TYR7 GLY8 GLY9 ASP10 PRO11 PRO15 TRP16 PRO17 CYS23 ALA24 GLY25 PHE245 ARG246 ALA247 THR248 GLN249 PRO250 ASN252
Carbonic Anhydrase XII	Compound	−7.8	636	THR6 TYR7 PHE8 CYS237 SER244 PRO245 ARG246 GLU247 MET248 ILE249 ASN250
Carbonic Anhydrase IX	AAZ	−6.7	333	ALA43 LEU44 LEU81 GLY82 PRO84 GLY85 ARG86 GLU87 TYR88 ARG89 SER124 THR125 ALA127 PHE128 ALA129 GLY136 PRO138
Carbonic Anhydrase XII	AAZ	−7.6	1764	THR6 TYR7 PHE8 GLY9 PRO10 ASP11 GLY12 GLU13 ASN14 SER15 CYS23 GLY24 GLY25 LEU26 LEU27 GLN28 GLN256 LYS257 PHE258 ASP259

**Figure 8:**
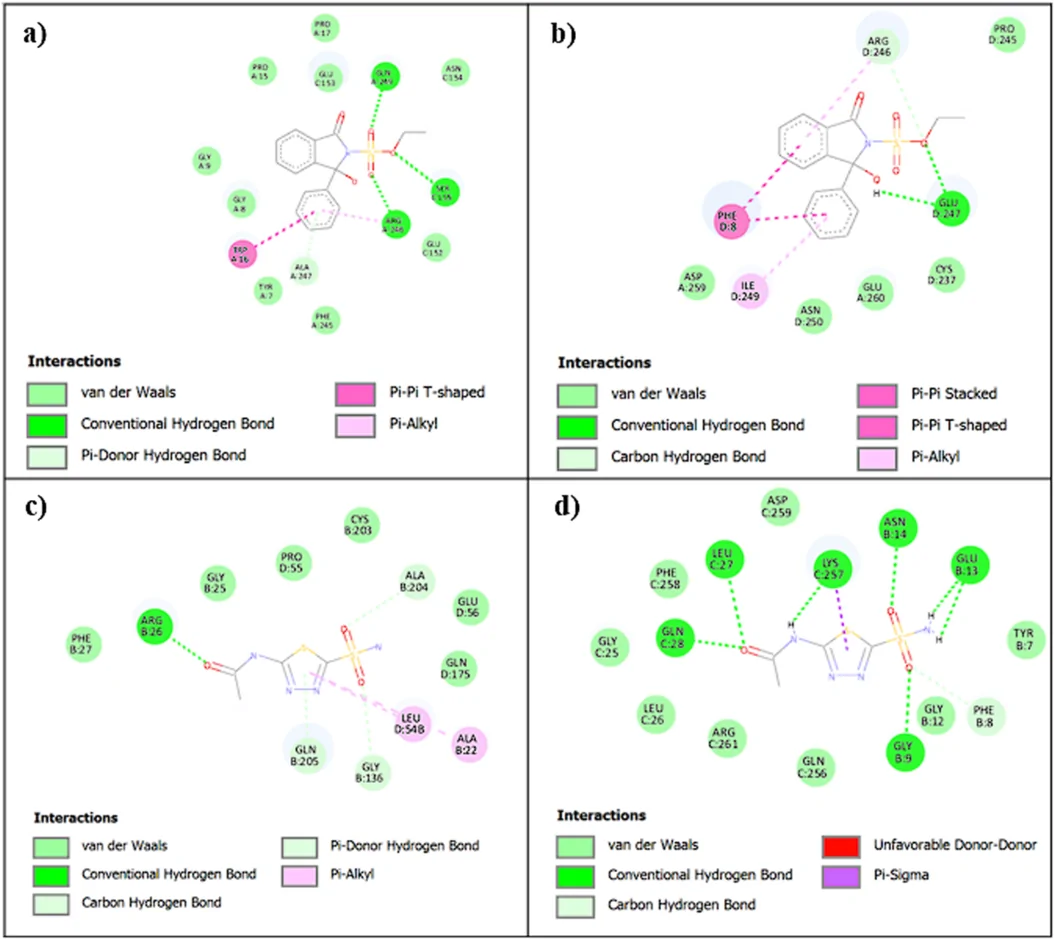
2D interaction diagram of intermolecular interactions depicted using Discovery Studio Visualizer, compound ligand and Carbonic Anhydrase IX (a); compound ligand and Carbonic Anhydrase XII (b); acetazolamide and Carbonic Anhydrase IX (c); acetazolamide and Carbonic Anhydrase XII (d). Dashed lines represent the different interactions and the interaction type. Colored circles represent residues with their three‐letter code, chain identifier and residue number.

**Figure 9:**
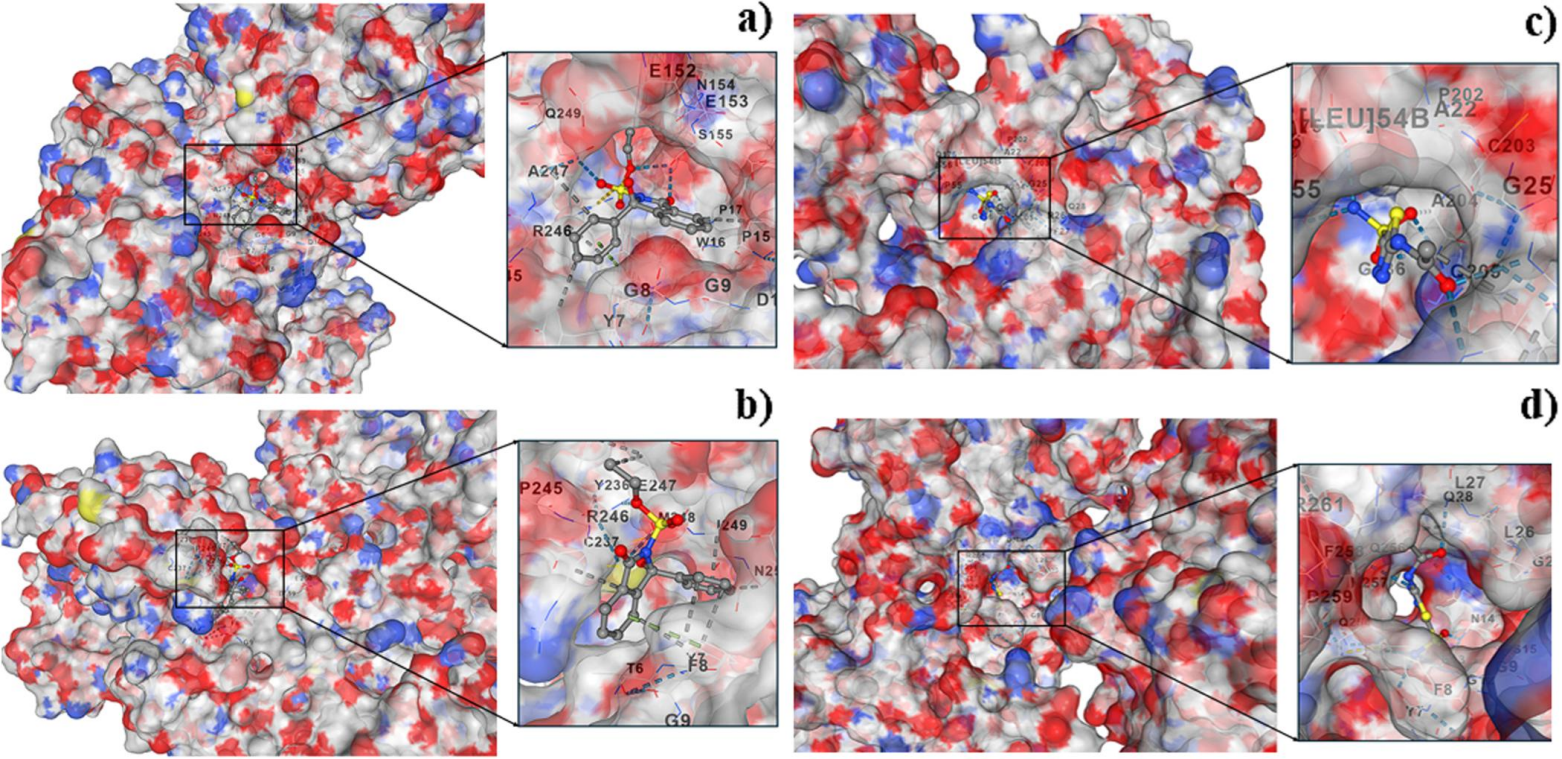
Visual representation of molecular docking interactions for compound and Carbonic Anhydrase IX (a), compound and Carbonic Anhydrase XII (b), acetazolamide and Carbonic Anhydrase IX (c), and acetazolamide and Carbonic Anhydrase XII (d).

## 4 Conclusion

In this study, ethyl 1‐hydroxy‐3‐oxo‐1‐phenylisoindoline‐2‐sulfonate exhibited potent inhibitory activity against the isolated tumor‐associated carbonic anhydrases hCA IX and hCA XII. Molecular docking further supported favorable predicted interactions with both isoforms. In MCF‐7 cells, the compound produced concentration‐dependent reductions in metabolic activity and membrane integrity and altered the immunofluorescence profiles of Caspase‐3, LC3B, GPX4, and TFRC, indicating the involvement of multiple cell‐death‐associated processes. The evaluation in nontumorigenic mammary epithelial cells also contributed to the preliminary assessment of the cellular selectivity and safety profile of the compound. However, due to the cellular experiments were conducted under normoxic conditions and cellular hCA IX/hCA XII expression and target engagement were not directly measured, the observed cytotoxicity cannot be attributed specifically to hCA IX inhibition. The cellular responses may involve hCA XII inhibition, CA‐independent mechanisms, or interactions with additional molecular targets. Overall, the potent enzyme‐inhibitory activity and the observed cellular effects support compound as a promising scaffold for further investigation. Future studies using aggressive breast cancer models, including MDA‐MB‐231 cells under normoxic and hypoxic conditions, together with direct measurements of cellular hCA IX/hCA XII expression, activity, and isoform‐specific target engagement, will help clarify its mechanism of action and translational potential.

## Author Contributions

**Funda Keteci:** writing draft, conceptualization, methodology, formal analysis, data curation. **Songul Bayrak:** writing – review and editing draft, methodology, validation, formal analysis, data curation. **Ufuk Atmaca:** investigation methodology, formal analysis, data curation. **Mustafa Özkaraca:** methodology, formal analysis. **Azada Aliyeva:** methodology, formal analysis. **Buket Bakan:** writing – reviewing draft, supervision, methodology, validation, formal analysis, data curation. All authors read and approved the manuscript.

## Funding

The authors have nothing to report.

## Ethics Statement

The authors have nothing to report.

## Conflicts of Interest

The authors declare no conflicts of interest.

## Data Availability

The data sets used and/or analyzed during the current study are available from the corresponding author on reasonable request.

## References

[1] L. P. Lin , P. Yuan , N. Jiang , et al., “Gene‐Inspired Mycosynthesis of Skeletally New Indole Alkaloids,” Organic Letters 17 (2015): 2610–2613. 10.1021/acs.orglett.5b00882 25985278

[2] M. G. Ciulla and K. Kumar , “The Natural and Synthetic Indole Weaponry Against Bacteria,” Tetrahedron Letters 59 (2018): 3223–3233.

[3] L. Meng , Q. Guo , Y. Liu , et al., “Indole Alkaloid Sulfonic Acids From an Aqueous Extract of *Isatis indigotica* Rroots and Their Antiviral Activity,” Acta Pharmaceutica Sinica B 7 (2017): 334–341. 10.1016/j.apsb.2017.04.003 PMC5430897 28540170

[4] W. Xu , D. J. Gavia , and Y. Tang , “Biosynthesis of Fungal Indole Alkaloids,” Natural Products Reports 31 (2014): 1474–1487. 10.1039/c4np00073k PMC4162825 25180619

[5] Z. Köksal , Z. Alım , S. Bayrak , İ. Gülçin , and H. Özdemir , “Investigation of the Effects of Some Sulfonamides on Acetylcholinesterase and Carbonic Anhydrase Enzymes,” Journal of Biochemical and Molecular Toxicology 33 (2019): 22300, 10.1002/jbt.22300. 30811749

[6] S. Bayrak , S. Gerni , C. Ozturk , B. Bakan , S. Elmas , and A. Aliyeva , “In Vitro Evaluation of Flavonoids for Enzyme Inhibition, Antioxidant, Antimicrobial and Anticancer Properties With Molecular Docking Insights,” Food Biophysics 20 (2025): 148.

[7] P. Begines , A. Bonardi , S. Giovannuzzi , et al., “Synthesis and Investigation of Selective Human Carbonic Anhydrase IX, XII Inhibitors Using Coumarins Bearing a Sulfonamide or Biotin Moiety,” Chemico‐Biological Interactions 404 (2024): 111284. 10.1016/j.cbi.2024.111284 39442681

[8] N. Chauhan , N. Yadav , P. Kumar , S. Kumar , and R. Aggarwal , “Exploring the SAR of 1,2,3‐Triazoles as Tumor‐Associated Carbonic Anhydrases IX and XII Inhibitors for Anticancer Applications,” Archiv der Pharmazie 358 (2025): 70041, 10.1002/ardp.70041. 40629941

[9] A. Angeli , F. Carta , and C. T. Supuran , “Carbonic Anhydrases: Versatile and Useful Biocatalysts in Chemistry and Biochemistry,” Catalysts 10 (2020): 1008.

[10] M. Palma , “Advancing Breast Cancer Treatment: The Role of Immunotherapy and Cancer Vaccines in Overcoming Therapeutic Challenges,” Vaccines 13 (2025): 344. 10.3390/vaccines13040344 PMC12030785 40333213

[11] Y. S. Yazıcıoğlu , Ş. Elmas , Z. Kılıç , et al., “Synthesis of Novel Isoindolinones: Carbonic Anhydrase Inhibition Profiles, Antioxidant Potential, Antimicrobial Effect, Cytotoxicity and Anticancer Activity,” Journal of Biochemical and Molecular Toxicology 39 (2025): 70261, 10.1002/jbt.70261. PMC11995831 40227019

[12] R. Verpoorte , Y. H. Choi , N. R. Mustafa , and H. K. Kim , “Metabolomics: Back to Basics,” Phytochemistry Reviews 7 (2008): 525–537.

[13] S. Mert , Y. Demir , Y. Sert , R. Kasımoğulları , and İ. Gülçin , “Synthesis, Biological Evaluation and Molecular Docking of Novel Pyrazole Derivatives as Multitarget Acetylcholinesterase and Carbonic Anhydrase Inhibitors,” Journal of Molecular Structure 1319 (2025): 139472.

[14] C. Öztürk , E. Kalay , S. Gerni , et al., “Sulfonamide Derivatives With Benzothiazole Scaffold: Synthesis and Carbonic Anhydrase I–II Inhibition Properties,” Biotechnology and Applied Biochemistry 71 (2024): 223–231. 10.1002/bab.2534 37964505

[15] C. Öztürk , N. Balci , O. N. Aslan , and E. Kalay , “Novel Sulfonylhydrazones With Sulfonate Ester Framework: Promising Dual Inhibitors of AChE and hCAs,” Biotechnology and Applied Biochemistry 72 (2025): 1752–1762. 10.1002/bab.2780 PMC12687696 40320825

[16] F. Keteci and B. Bakan , “Comparative Analysis of Bisphenol A and Its Derivatives (BPF and BPS) on Oxidative Injury and Apoptosis in Dermal Fibroblasts,” Journal of Applied Toxicology 45 (2025): 2735–2745. 10.1002/jat.4931 41013979

[17] B. Bakan , M. Tokmak , E. Toraman , Ş. Elmas , A. S. Mendil , and M. Özkaraca , “Toxicological Investigation and Gene‐Related Molecular Interactions of Polytetrafluoroethylene Nanoparticles (PTFE NPs) on Human Breast Cells,” Molecular Biology Reports 52 (2025): 914. 10.1007/s11033-025-11033-w 40960682

[18] S. Güngör and B. Bakan , “Novel Synthesized, Characterization and In Vitro Toxicological Evaluation of Amino Acid‐Based‐Cerium Oxide Nanoparticles (CeO_2_ NPs),” Drug and Chemical Toxicology 48 (2025): 697–708. 10.1080/01480545.2025.2484461 40162520

[19] H. M. Berman , “The Protein Data Bank,” Nucleic Acids Research 28 (2000): 235–242. 10.1093/nar/28.1.235 PMC102472 10592235

[20] Y. Liu , X. Yang , J. Gan , S. Chen , Z. X. Xiao , and Y. Cao , “CB‐Dock2: Improved Protein–Ligand Blind Docking by Integrating Cavity Detection, Docking and Homologous Template Fitting,” Nucleic Acids Research 50 (2022): W159–W164. 10.1093/nar/gkac394 PMC9252749 35609983

[21] S. Si Baroroh , U. M.Biotek , Z. S. Muscifa , W. Destiarani , et al., “Molecular Interaction Analysis and Visualization of Protein‐Ligand Docking Using Biovia Discovery Studio Visualizer,” Indonesian Journal of Computational Biology 2 (2023): 22.

[22] E. F. Pettersen , T. D. Goddard , C. C. Huang , et al., “UCSF Chimera—A Visualization System for Exploratory Research and Analysis,” Journal of Computational Chemistry 25 (2004): 1605–1612. 10.1002/jcc.20084 15264254

[23] C. Kakakhan , C. Türkeş , Ö. Güleç , et al., “Exploration of 1,2,3‐Triazole Linked Benzenesulfonamide Derivatives as Isoform Selective Inhibitors of Human Carbonic Anhydrase,” Bioorganic & Medicinal Chemistry 77 (2023): 117111. 10.1016/j.bmc.2022.117111 36463726

[24] G. Giuntini , S. Monaci , Y. Cau , M. Mori , A. Naldini , and F. Carraro , “Inhibition of Melanoma Cell Migration and Invasion Targeting the Hypoxic Tumor Associated CAXII,” Cancers 12 (2020): 3018. 10.3390/cancers12103018 PMC7602957 33080820

[25] U. Atmaca , R. Saglamtas , Y. Sert , et al., “Metal‐Free Synthesis via Intramolecular Cyclization, Enzyme Inhibition Properties and Molecular Docking of Novel Isoindolinones,” ChemistrySelect 8, ahead of print, March 6, 2023, 10.1002/slct.202204578.

[26] B. Z. Kurt , A. Dag , B. Doğan , et al., “Synthesis, Biological Activity and Multiscale Molecular Modeling Studies of Bis‐Coumarins as Selective Carbonic Anhydrase IX and XII Inhibitors With Effective Cytotoxicity Against Hepatocellular Carcinoma,” Bioorganic Chemistry 87 (2019): 838–850. 10.1016/j.bioorg.2019.03.003 31003041

[27] S.‐H. Lee and J. R. Griffiths , “How and Why Are Cancers Acidic? Carbonic Anhydrase IX and the Homeostatic Control of Tumour Extracellular pH,” Cancers 12 (2020): 1616. 10.3390/cancers12061616 PMC7352839 32570870

[28] H. Nadaroglu , A. A. Gungor , Ö. Gundogdu , N. H. Kishali , B. Sever , and M. D. Altintop , “Investigation of the Inhibitory Effects of Isoindoline‐1,3‐dion Derivatives on hCA‐I and hCA‐II Enzyme Activities,” Journal of Molecular Structure 1197 (2019): 386–392.

[29] M. Awadallah , M. Idle , K. Patel , and D. Kademani , “Management Update of Potentially Premalignant Oral Epithelial Lesions,” Oral Surgery, Oral Medicine, Oral Pathology and Oral Radiology 125 (2018): 628–636. 10.1016/j.oooo.2018.03.010 29656948

[30] S. Gündoğdu , C. Türkeş , M. Arslan , Y. Demir , and Ş. Beydemir , “New Isoindole‐1,3‐dione Substituted Sulfonamides as Potent Inhibitors of Carbonic Anhydrase and Acetylcholinesterase: Design, Synthesis, and Biological Evaluation,” ChemistrySelect 4 (2019): 13347–13355.

[31] S. Ivanov , S.‐Y. Liao , A. Ivanova , et al., “Expression of Hypoxia‐Inducible Cell‐Surface Transmembrane Carbonic Anhydrases in Human Cancer,” American Journal of Pathology 158 (2001): 905–919. 10.1016/S0002-9440(10)64038-2 PMC1850340 11238039

[32] D. Vergara , S. Ravaioli , E. Fonzi , et al., “Carbonic Anhydrase XII Expression Is Modulated During Epithelial Mesenchymal Transition and Regulated Through Protein Kinase C Signaling,” International Journal of Molecular Sciences 21 (2020): 715. 10.3390/ijms21030715 PMC7037142 31979064

[33] Y. Li , H. Wang , C. Tu , K. T. Shiverick , D. N. Silverman , and S. C. Frost , “Role of Hypoxia and EGF on Expression, Activity, Localization and Phosphorylation of Carbonic Anhydrase IX in MDA‐MB‐231 Breast Cancer Cells,” Biochimica et Biophysica Acta (BBA) – Molecular Cell Research 1813 (2011): 159–167. 10.1016/j.bbamcr.2010.09.018 PMC3014402 20920536

[34] F. E. Lock , P. C. McDonald , Y. Lou , et al., “Targeting Carbonic Anhydrase IX Depletes Breast Cancer Stem Cells Within the Hypoxic Niche,” Oncogene 32 (2013): 5210–5219. 10.1038/onc.2012.550 23208505

[35] S. Mesci , M. Gül , S. Eryılmaz , T. Lis , S. Szafert , and T. Yıldırım , “Antioxidant and Cytotoxic Activity of Isoindole Compounds in Breast Cancer Cells (MCF‐7),” Journal of Molecular Structure 1217 (2020): 128366.

[36] A. Tan , A. S. Yaglioglu , N. H. Kishali , E. Sahin , and Y. Kara , “Evaluation of Cytotoxic Potentials of Some Isoindole‐1,3‐dione Derivatives on HeLa, C6 and A549 Cancer Cell Lines,” Medicinal Chemistry 16 (2020): 69–77. 10.2174/1573406415666181206115638 30520382

[37] A. Tan , S. Kizilkaya , U. Kelestemur , A. Akdemir , and Y. Kara , “The Synthesis, Anticancer Activity, Structure‐Activity Relationships and Molecular Modelling Studies of Novel Isoindole‐1,3(2H)‐dione Compounds Containing Different Functional Groups,” Anti‐Cancer Agents in Medicinal Chemistry 20 (2020): 1368–1378. 10.2174/1871520620666200410080648 32275494

[38] I. Ledaki , A. McIntyre , S. Wigfield , et al., “Carbonic Anhydrase IX Induction Defines a Heterogeneous Cancer Cell Response to Hypoxia and Mediates Stem Cell‐Like Properties and Sensitivity to HDAC Inhibition,” Oncotarget 6 (2015): 19413–19427. 10.18632/oncotarget.4989 PMC4637295 26305601

[39] N. K. Tafreshi , M. C. Lloyd , J. B. Proemsey , et al., “Evaluation of CAIX and CAXII Expression in Breast Cancer at Varied O_2_ Levels: CAIX Is the Superior Surrogate Imaging Biomarker of Tumor Hypoxia,” Molecular Imaging and Biology 18 (2016): 219–231. 10.1007/s11307-015-0885-x PMC4754166 26276155

[40] A. Janoniene , L. Mazutis , D. Matulis , and V. Petrikaite , “Inhibition of Carbonic Anhydrase IX Suppresses Breast Cancer Cell Motility at the Single‐Cell Level,” International Journal of Molecular Sciences 22 (2021): 11571. 10.3390/ijms222111571 PMC8584155 34769000

[41] P. Biswas , D. Dey , P. K. Biswas , et al., “A Comprehensive Analysis and Anti‐Cancer Activities of Quercetin in ROS‐Mediated Cancer and Cancer Stem Cells,” International Journal of Molecular Sciences 23 (2022): 11746. 10.3390/ijms231911746 PMC9569933 36233051

[42] S. H. Mousavi , J. Tavakkol‐Afshari , A. Brook , and I. Jafari‐Anarkooli , “Direct Toxicity of Rose Bengal in MCF‐7 Cell Line: Role of Apoptosis,” Food and Chemical Toxicology 47 (2009): 855–859. 10.1016/j.fct.2009.01.018 19271285

[43] W. Zhang , Y. Liu , Y. Liao , C. Zhu , and Z. Zou , “GPX4, Ferroptosis, and Diseases,” Biomedicine & Pharmacotherapy 174 (2024): 116512. 10.1016/j.biopha.2024.116512 38574617

[44] S. J. Dixon , K. M. Lemberg , M. R. Lamprecht , et al., “Ferroptosis: An Iron‐Dependent Form of Nonapoptotic Cell Death,” Cell 149 (2012): 1060–1072. 10.1016/j.cell.2012.03.042 PMC3367386 22632970

[45] X.‐Y. Meng , H.‐X. Zhang , M. Mezei , and M. Cui , “Molecular Docking: A Powerful Approach for Structure‐Based Drug Discovery,” Current Computer Aided‐Drug Design 7 (2011): 146–157. 10.2174/157340911795677602 PMC3151162 21534921

[46] N. S. Pagadala , K. Syed , and J. Tuszynski , “Software for Molecular Docking: A Review,” Biophysical Reviews 9 (2017): 91–102. 10.1007/s12551-016-0247-1 PMC5425816 28510083

[47] D. B. Kitchen , H. Decornez , J. R. Furr , and J. Bajorath , “Docking and Scoring in Virtual Screening for Drug Discovery: Methods and Applications,” Nature Reviews Drug Discovery 3 (2004): 935–949. 10.1038/nrd1549 15520816

[48] L. Ferreira , R. Dos Santos , G. Oliva , and A. Andricopulo , “Molecular Docking and Structure‐Based Drug Design Strategies,” Molecules 20 (2015): 13384–13421. 10.3390/molecules200713384 PMC6332083 26205061

[49] I. R. Hardcastle , S. U. Ahmed , H. Atkins , et al., “Small‐Molecule Inhibitors of the MDM2‐p53 Protein−Protein Interaction Based on an Isoindolinone Scaffold,” Journal of Medicinal Chemistry 49 (2006): 6209–6221. 10.1021/jm0601194 17034127

[50] V. Alterio , A. Di Fiore , K. D'Ambrosio , C. T. Supuran , and G. De Simone , “Multiple Binding Modes of Inhibitors to Carbonic Anhydrases: How to Design Specific Drugs Targeting 15 Different Isoforms?,” Chemical Reviews 112 (2012): 4421–4468. 10.1021/cr200176r 22607219

[51] W. M. Eldehna , M. F. Abo‐Ashour , E. Berrino , et al., “SLC‐0111 Enaminone Analogs, 3/4‐(3‐Aryl‐3‐oxopropenyl) Aminobenzenesulfonamides, as Novel Selective Subnanomolar Inhibitors of the Tumor‐Associated Carbonic Anhydrase Isoform Ix,” Bioorganic Chemistry 83 (2019): 549–558. 10.1016/j.bioorg.2018.11.014 30471577

[52] S. G. Nerella , P. Singh , M. Arifuddin , and C. T. Supuran , “Anticancer Carbonic Anhydrase Inhibitors: A Patent and Literature Update 2018‐2022,” Expert Opinion on Therapeutic Patents 32 (2022): 833–847. 10.1080/13543776.2022.2083502 35616541

[53] M. B. Haider , R. Kanwal , A. Ahmed , et al., “Design, Synthesis, Characterization and Exploration of Biological Efficacy of Iminothiazoline Sulfonamide Hybrids as Efficient Inhibitor of Carbonic Anhydrase,” Journal of Molecular Structure 1338 (2025): 142331.

[54] Z. Zhang , H. Yang , Y. Zhong , et al., “Synthesis, Molecular Docking Analysis, and Biological Evaluations of Saccharide‐Modified Sulfonamides as Carbonic Anhydrase IX Inhibitors,” International Journal of Molecular Sciences 22 (2021): 13610. 10.3390/ijms222413610 PMC8704611 34948406

